# Mkrn3 functions as a novel ubiquitin E3 ligase to inhibit Nptx1 during puberty initiation

**DOI:** 10.18632/oncotarget.19347

**Published:** 2017-07-18

**Authors:** Huifang Liu, Xiangxin Kong, Fengling Chen

**Affiliations:** ^1^ Department of Endocrinology, Shanghai Ninth People’s Hospital, Shanghai Jiao Tong University School of Medicine, Shanghai, China

**Keywords:** puberty, Mkrn3, Nptx1, ubiquitination, hypothalamus

## Abstract

Central precocious puberty (CPP) is attributed to the disorder of some trigger factors those can activate the hypothalamic-pituitary-gonadal axis controlled by GnRH neurons. Many recent studies reveal one of those trigger factors, Makorin ring finger protein 3 (Mkrn3), whose loss-of-function mutations are implicated in CPP. Although Mkrn3 contained zinc Ring finger domain is considered as a putative E3 ubiquitin ligase, its actual function is never reported. Here, our results demonstrated that in mice hypothalamus before and when puberty initiated, Mkrn3 expressed the reversed tendency with Nptx1, which is an important secreted protein for neuron development. Furthermore, our data manifested that Mkrn3 interacted and suppressed Nptx1 activity. And the Ring finger domain of Mkrn3 contained was determined to be essential for binding with Nptx1 for its polyubiquitination during the puberty initiation. Our study shed light on the molecular insights into the function of Mkrn3 in the events of puberty initiation.

## INTRODUCTION

Puberty is the physiological transition stage between childhood to adulthood. From the aspect of neuroendocrine, the enhanced and sustained pulsatile release of gonadotropin-releasing hormone (GnRH) in hypothalamus activates the hypothalamic-pituitary-gonadal axis and contributes to mammalian puberty initiation. The premature reactivation of this axis at the inappropriate chronological age results in central precocious puberty (CPP) [1]. Multiple disorders of genetic, nutritional and environmental factors driven by GnRH construct a complex interaction network and play a crucial role in determining the onset of puberty. The studies on the neuroendocrine mechanisms of CPP provided the evidence of stimulatory or inhibitory effect on GnRH neurons governed by many genes [2]. Recent studies also have been conducted to explore the genetic causes for CPP [3–5]. However, further functions of those GnRH targets were still less studied.

Makorin ring finger protein 3 (Mkrn3) is a novel gene identified from human families with CPP using whole-exome sequencing [6]. So far, 27 missense and frameshift mutations have been discovered from pedigree investigation. The loss-of-function mutations of Mkrn3 are highly correlated with CPP, which implies that Mkrn3 is important for puberty initiation. Mkrn3 protein has 4 zinc finger domains namely 3 C3H1 motifs and 1 C3H4 Ring finger with presumed E3 ubiquitin ligase activity [7]. Although Mkrn3 is postulated to be an inhibit stimulator of GnRH secretion [8], however, the molecular mechanism of Mkrn3 effect on GnRH network still remains unclear. Nevertheless, the structure of Ring finger domain within Mkrn3 protein gives us a clue that Mkrn3 may exert as an E3 ubiquitin ligase to suppress GnRH activity.

One of our interested studies includes one secreted protein, neural pentraxin-1 precursor (Nptx1), which is observed highly expressed in hypothalamus when puberty onset from public databases (GDS3110, GDS3847) [9, 10] and plays an important role in neural differentiation [11]. However, most studies on Nptx1 mainly focus on the synapse formation and remodeling and the related diseases of neuronal damage or injury, but never on the sexual development. Based on this phenotype of Nptx1 spatial and temporal-specific expression in hypothalamus, we try to figure out the regulatory mechanism of Nptx1 during central puberty. Interestingly, we conducted immunoprecipitation to pull down Nptx1 in mice hypothalamus before and during puberty, and identified Mkrn3 as one of the binding proteins by mass spectrometry analysis (Supplementary Figure 1).

In this study, we illuminate the connection between Nptx1 and Mkrn3, investigate the function of Mkrn3, and try to provide the molecular insight into the complex regulatory network of Mkrn3 when puberty initiation.

## RESULTS

### The extracellular level of Nptx1 is highly secreted during the puberty

**Figure 1:**
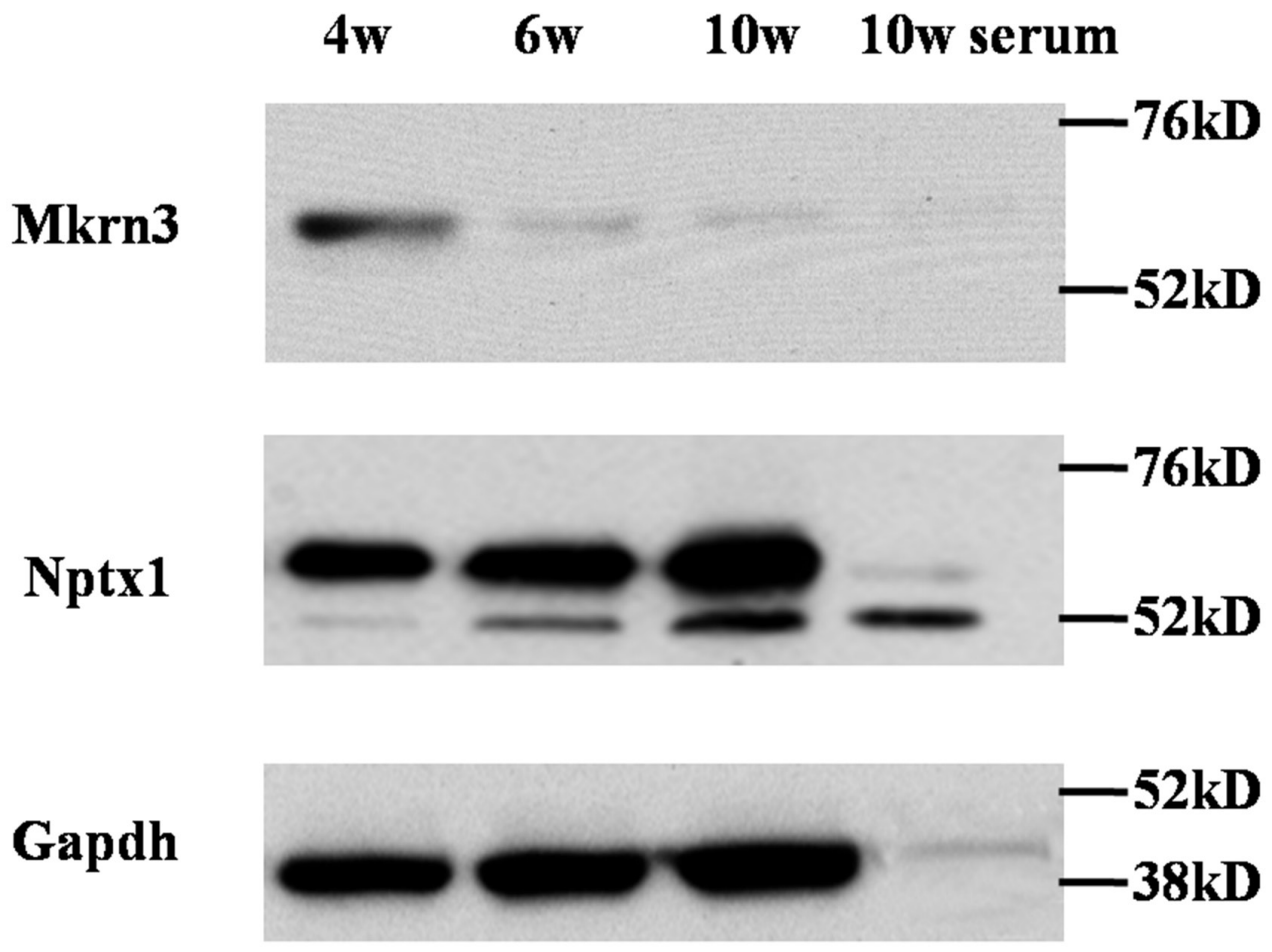
The expression of Mkrn3 and Nptx1 in hypothalamus of 4-, 6- and 10-week old female mice

The presence of low expression of Mkrn3 in hypothalamus and preoptic area at the postnatal 6^th^ week compared to the embryonic and neonatal mice is consistent with previous human study, which implies Mkrn3 exert inhibitory effect when puberty initiates [12]. To validate the expressed pattern of Mkrn3 and Nptx1 in hypothalamus, the expression of these two endogenous proteins were investigated in the whole tissue lysis of hypothalamus of 4-, 6- and 10- week old female mice (Figure 1, Supplementary Figure 2). Considering a secreted protein of Nptx1, precursor protein was usually synthesized in cytoplasm and secreted to extracellular matrix after the signal peptide removal. Therefore, the upper and bottom bands were Nptx1 protein with or without signal peptide, namely, intra- and extra-cellular Nptx1 respectively. We prepared primary antibody of Nptx1 against the N-terminal region (2-28) for WB assay and observed that the secreted Nptx1 was highly expressed in hypothalamus of 6- and 10- compared to 4-week old. Interestingly, the level of premature Nptx1 (upper band) actually was equal, which implied that Nptx1 was mainly regulated not by transcriptional but posttranslational modification during puberty. Conversely, we also observed Mkrn3 was sharply reduced in 6- and 10- compared to 4-week old hypothalamus, which was consistent with previous studies that Mkrn3 disappears after puberty initiates [12]. Taken together, the dynamic change of Nptx1 and Mkrn3 expression in hypothalamus of different stages of puberty suggests that Mkrn3 may play an inhibitory role in posttranslational regulation of Nptx1.

### The interaction between Nptx1 and Mkrn3 in hypothalamic neuron cells *in vivo*

**Figure 2:**
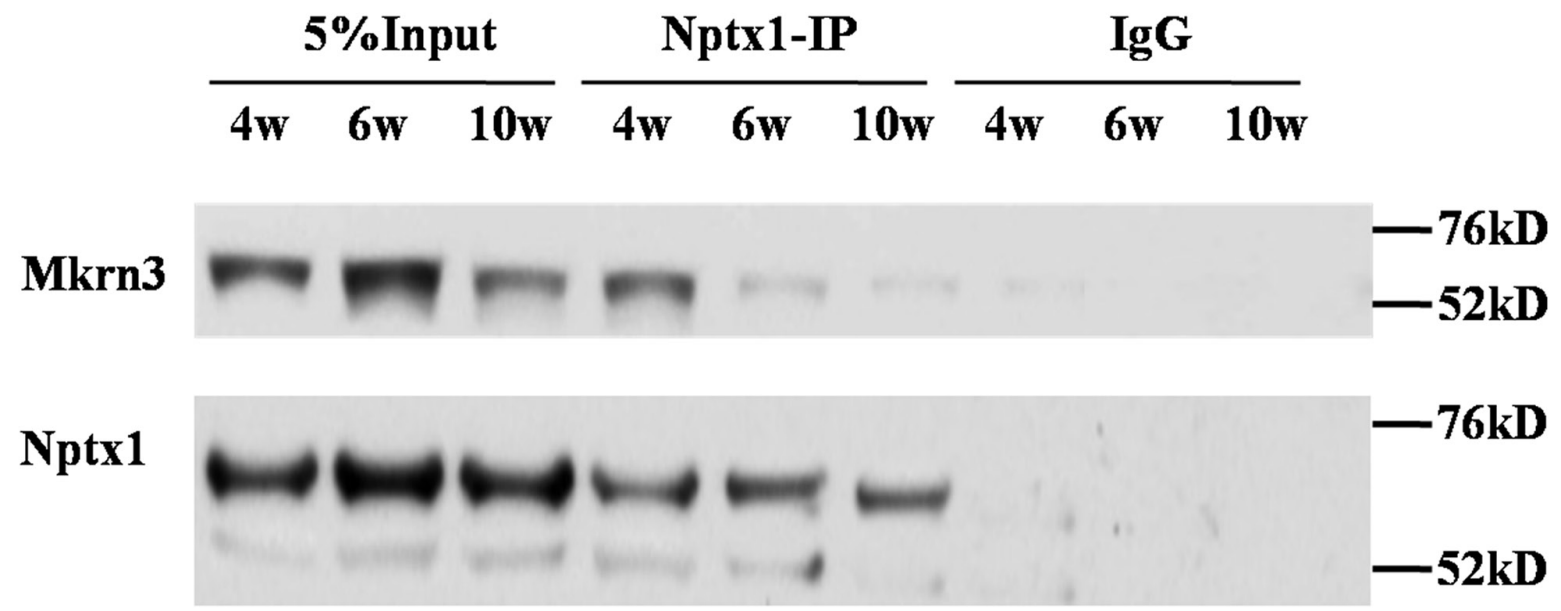
The interaction between Mkrn3 and Nptx1 in hypothalamus

Given our preliminary blue staining results identified by mass spectrometry (Supplementary Figure 1), the interaction between Nptx1 and Mkrn3 was further confirmed by co-IP. 3-week old mice received lateral cerebroventricular administration of Flag labeled Mkrn3 and sacrificed in 4-, 6- and 10-week old for IP investigation (Supplementary Figure 3). Owing to the unavailable commercial IP degree antibody of Mkrn3, we only conducted Nptx1-IP and detected wild type Mkrn3 (Figure 2). After the equal Nptx1 level normalization, Mkrn3 is observed apparently strong interaction in hypothalamus of 4-week old mice compared to 6- and 10-week, which is consistent with protein expression (Figure 1) that down-regulated Mkrn3 after puberty initiates results in the loss of interaction. In conclusion, we validate the substantial interaction between Nptx1 and Mkrn3 from mass spectrum data.

**Figure 3:**
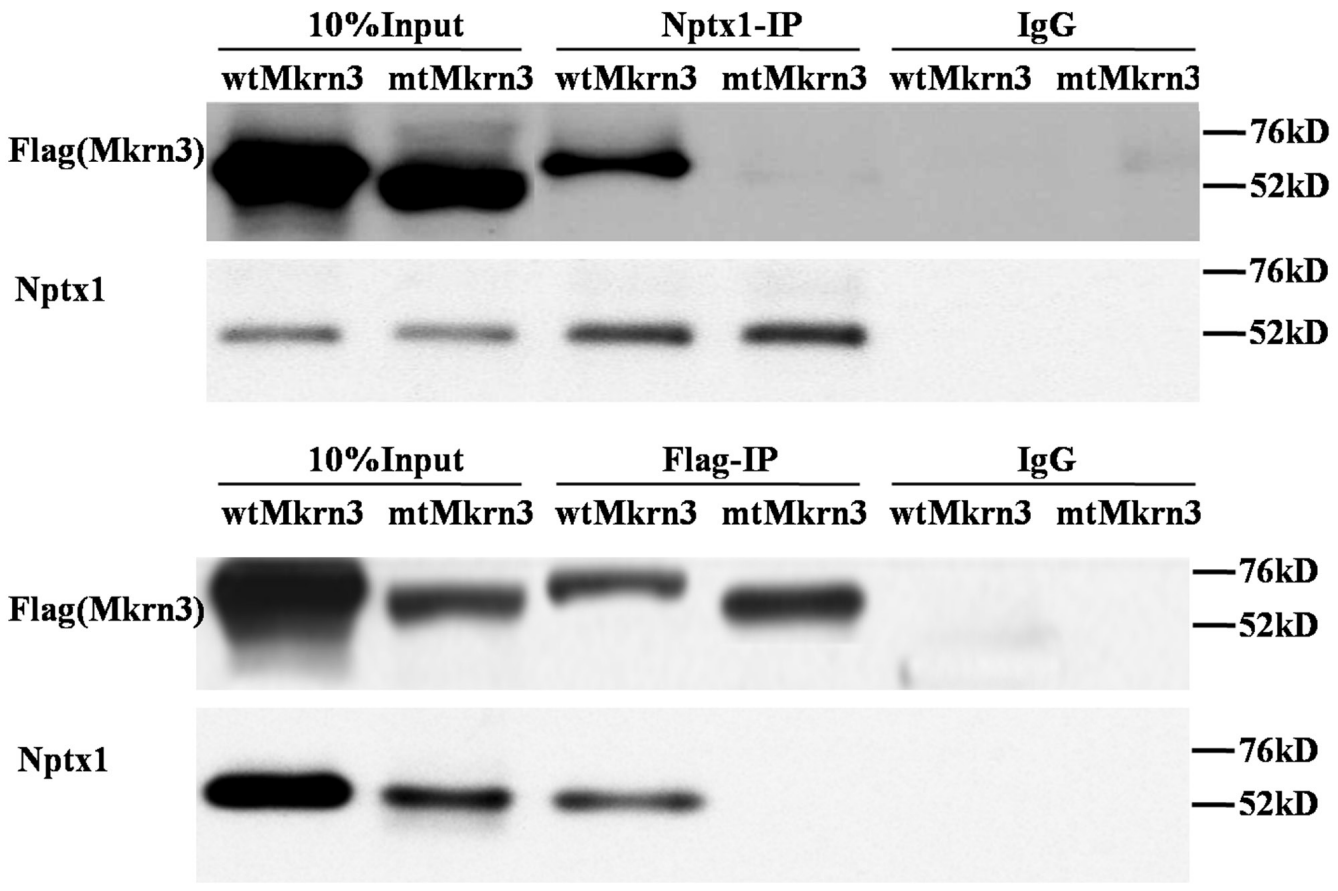
Ring finger of Mkrn3 essential for interaction with Nptx1

In the case of Mkrn3, we notice that only the first and final C3H1 and C3HC4 Ring finger domains are conserved in human and mouse species. Meanwhile, 40% missense mutations are discovered in C3HC4 Ring finger domain in familial investigation of CPP [6, 12–16]. Although the C3HC4 Ring finger domain of Mkrn3 is assumed as E3 ubiquitin ligase, the function, however, is never studied. We suspected this domain might be important for Nptx1interaction and regulation, and therefore constructed the exogenous vectors of Flag labeled wild type (wt) and mutant (mt) Mkrn3 without C3HC4 Ring finger motif (347-401), and transfected into mouse hypothalamus via lateral cerebral ventricle administration to detect Flag or Nptx1 level of hypothalamus of 4-week old mice to investigate the affinity of interaction using co-IP. Obviously, the presence of interaction between Nptx1 and mtMkrn3 failed to be observed compared to wtMkrn3 (Figure 3). Taken together, the C3HC4 Ring finger domain of Mkrn3 is essential for Nptx1 interaction and plays an important role for Nptx1 inhibition in juvenile period.

**Figure 4:**
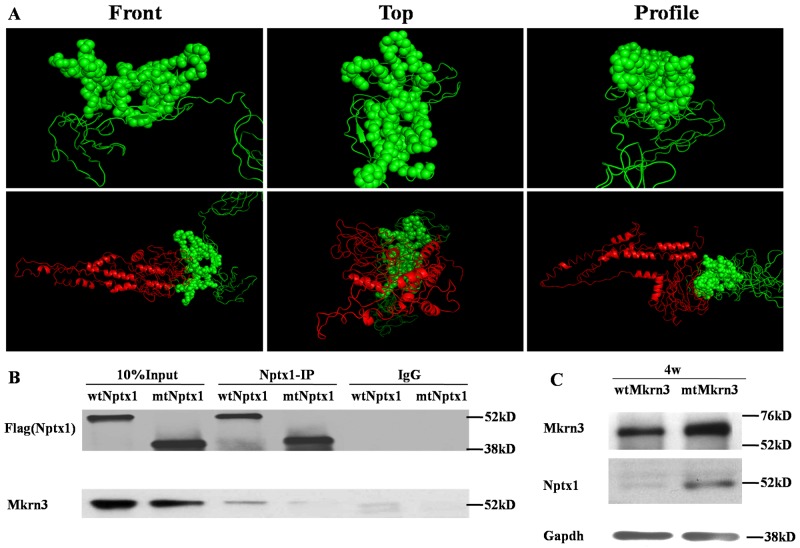
The interaction domain between Mkrn3 and Nptx1 **(A)** The homology modeling of the Ring finger of Mkrn3 (Upper) and molecular docking with Mkrn3 and Nptx1 (Bottom). Green and red cartoon represent Mkrn3 and Nptx1 respectively. Sphere is for highlighting the interacted domain of Mkrn3. **(B)** The interacted domain of Nptx1 with Mkrn3 confirmed by IP. **(C)** The regulatory effect of Ring finger domain of Mkrn3 on Nptx1.

Ring finger proteins, mostly, directly bind with their substrates [17]. Furthermore, we try to explore how the C3HC4 ring finger domain of Mkrn3 tethers with Nptx1. To this end, we did homologous modeling and molecular docking for Mkrn3 and Nptx1. Given the top credible models, the Ring finger and the C-terminus of Mkrn3 were supposed to form like a “sword hilt” and mainly bind with the coil residue (from 268-355) contained around 90 amino acids of Nptx1 (Figure 4A). According to the potential atoms of polar contact picked up from the docking model, we constructed the vector of Flag labeled mtNptx1 with 268-302 residues deletion and injected into lateral cerebroventricle for IP study in 4-week old mice hypothalamus. Compared to wtNptx1, mtNptx1 was observed less interaction with Mkrn3 (Figure 4B), which was consistent with our molecular docking study.

Besides the interaction between these two proteins was revealed, the function of their domain deletion was further confirmed. Because the inhibitory effect of Mkrn3 on Nptx1 mainly occurs in early juvenile, we focused on the hypothalamus of 4-week old mice, and investigated the expression of Nptx1 in the groups of wtMkrn3, mtMkrn3. The level of Nptx1 without signal peptide in wtMkrn3 group was obviously lower than mtMkrn3 group (Figure 4C), which indicates that the Ring figure domain of Mkrn3 is required for Nptx1 post-translational modification in early juvenile. Collectively, we revealed the domain found from docking model is essential for interaction and effective for Mkrn3 function in hypothalamus.

### The ubiquitination modulation of Nptx1 during puberty

**Figure 5:**
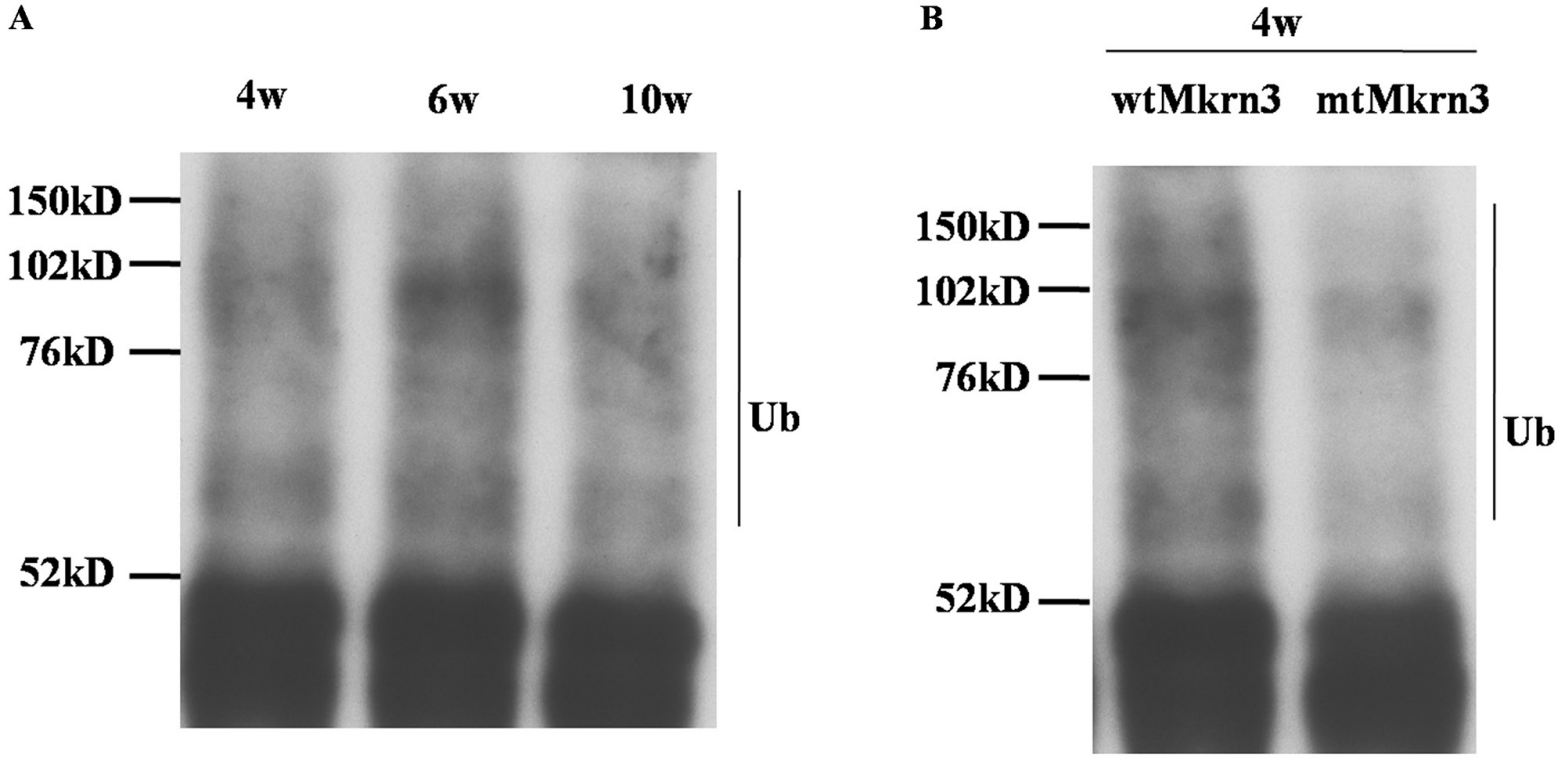
The polyubiquitination modification of Nptx1 by Mkrn3 **(A)** The polyubiquitination of Nptx1 in hypothalamus of 4-, 6- and 10-week old female mice. **(B)** The polyubiquitination of Nptx1 in 4-week old mice treated by wtMkrn3 and mtMkrn3 via lateral cerebroventricular administration.

The presence of Mkrn3 highly binding with Nptx1 via Ring finger motif as well as the low expression of Nptx1 in early juvenile period suggests that Nptx1 may be polyubiquitinated for degradation by Mkrn3. Therefore, endogenous Nptx1 protein in hypothalamus of 4-, 6- and 10-week old mice were pulled down and detected by ubiquitin antibody. The ladder bands of ubiquitination are observed in of 4-, 6- and 10-week old normal mice but no obvious difference (Figure 5A). While the polyubiquitination level of Nptx1 in the hypothalamus of the wtMkrn3 groups is higher than the mtMkrn3 groups in 4-week old mice (Figure 5B), which are both consistent with our hypothesis that Mkrn3 contributes the polyubiquitination of Nptx1 to degrade in early juvenile and the loss of Mkrn3 leads to Nptx1 high expressed from prepuberty to postpuberty.

## DISCUSSION

Although the genetic defect of Mkrn3 has been widely demonstrated to be closely related to the morbidity rate of CPP in sporadic and familial cases, the function of Mkrn3 is not yet completely explored. Mkrn3 is highly expressed in nervous system when early embryo and juvenile period but declines immediately before puberty onset and reaches very low level in adult life [6, 18], implying an inhibitory role of Mkrn3 on GnRH secretion [19, 20].

Our study discovered Mkrn3 might interact with Nptx1. Furthermore, Nptx1 regulated neural lineage commitment and specification as a niche supporter [11] and was reported to express robustly in melanin-concentrating hormone neurons of lateral hypothalamic area and zona incertafor synaptic formation [21, 22]. Nevertheless, it is hard to figure out the definite confine between arcuate nucleus and lateral hypothalamic area or zona incerta [23]. To date, no direct evidence shows Nptx1 exerts in GnRH neurons activity, but our result of dynamic change of Nptx1 expression in different stage of hypothalamus (Figure 1) indicates that Nptx1, one of the earliest activated extracellular signals, can respond upon GnRH neurons induction when puberty initiates.

Our data determine that Mkrn3 interacted with Nptx1 mainly via C3H4 Ring finger (Figure 2, 3), which is consistent with the molecular docking (Figure 4A). Mkrn gene family encodes ribonucleoprotein with a particular array of zinc finger motifs [24, 25]. Mkrn3 has a centrally located Ring finger motif that is a signature domain for E3 ligases, a category of enzymes mediating the transfer of ubiquitin from an E2 ubiquitin-conjugating enzyme to target protein substrates. Ubiquitination lead to various effects on the protein substrate, varying from the proteasome dependent proteolysis to regulation of protein function and localization [26]. Compared to the residues having the reported missense mutations in human MKRN3, the binding domain of Mkrn3 interacted with Nptx1 in this study do not completely overlap with these hot spot mutations but are very close to them (Supplementary Figure 4). We speculated that any atoms within this conserved motif may all contribute to maintaining the function of Mkrn3 even if the direct binding sites were not mutated.

Mkrn3 interacts with Nptx1 via Ring finger, a putative E3 ubiquitin ligase domain and has ability to suppress Nptx1 expression (Figure 1, 4C), implied that Nptx1 is ubiquitinated by Mkrn3. Nevertheless, the bands of polyubiquitination are observed but no difference before and after puberty (Figure 5A). Because the ubiquitination of Nptx1 has never been reported so far, we suspect that Nptx1, as a secreted protein, might be polyubiquitinated not only by Mkrn3 but also by other E3 ubiquitin ligase when and after puberty initiates. Interestingly, it is apparent that this polyubiquitination of Nptx1 in hypothalamus of 4-week old mice is higher in wtMkrn3 group than the mtMkrn3 group (Figure 5B), which means that in early juvenile, Mkrn3 mainly contribute to Nptx1 ubiquitinated modification for protein degradation in cytoplasm. Furthermore, our molecular docking model suggests that the key lysines of Nptx1 for polyubiquitination might be within the domain interacted with Mkrn3, but we did not find any difference of polyubiquitination level between wtNptx1 and mtNptx1 in 4-week old mice (data not shown). We have to admit that our docking model may not be perfect. Firstly, Nptx1 may actually have more lysines that can be ubiquitinated by Mkrn3 than what we found in our model. Since the interacted region (268-355) of Nptx1 called pentraxin protein domain (Figure 4B) is demonstrated to involve in mediating the uptake of synaptic macromolecules and playing a role in synaptic plasticity [27, 28], which suggests that this important functional domain of Nptx1 is the exact catalytic target attacked by Mkrn3. However, the E2-conjugating enzyme in this case is not illuminated yet. Mkrn3 may be the one that can recognize Nptx1 specifically and recruit E2-conjugating enzyme to form the complex. Once the new candidate proteins are discovered, the detailed information of ubiquitination should be further described. Finally, the reason why Mkrn3 vanish promptly from prepuberty and sustain in the entire adult life still need to be further studied. We raise more questions on the biological action and function of Mkrn3 when puberty initiates.

Now our results conclude that Mkrn3 exerts inhibitory effect upon Nptx1 via polyubiquitination during early juvenile. The unknown mechanism results in Mkrn3 silencing when puberty initiates so that GnRH neuron triggers such as Nptx1 start to be activated in succession. However, Mkrn3 loss of function derived from mutation leads to deficiency of inhibitory effect in early juvenile, and those triggers are activated earlier for CPP onset. And Ring finger motif of Mkrn3 is determined to be important to regulate polyubiquitination of Nptx1 before puberty. Additionally, since Mkrn3 has been determined to contribute to the suppression of trigger proteins maturation for puberty delay in childhood, the genetic treatment for compensation of correct Mkrn3 may be a potential approach for CPP therapy. However, the multiple types and amounts of zinc finger motifs in makorin proteins determine the multiple cellular functions of those proteins, which implies that it is not an appropriate strategy to relieve CPP through up-regulation of Mkrn family or other proteins contained Ring finger motif. We speculate Mkrn3 protein structure has other unique and important functional domains, which can help recognize the GnRH trigger factors specifically. And the complete Mkrn3 protein function still need to be further studied.

## MATERIALS AND METHODS

### Animals

All the procedures were approved by the Institutional Animal Care and Use Committee of Shanghai, China. The outbred female ICR mice were used in this study. Animals were fed with food and water *ad libitum* freely. Protocols were conducted to minimize pain and discomfort to the animals. 10 female animals in each group with postnatal 4-, 6- and 10- week old representing juvenile, puberty and postpuberty respectively were randomly assigned and intraperitoneal anesthetized by chloral hydrate (0.3 mL/100 g) then guillotined. Brain tissues were harvested quickly, frozen on dry ice. Hypothalamus was dissected as a rectangle from the anterior optic chiasm to posterior mamillary bodies and two coronal sides with 2mm depth [29] and stored in -80^o^C for next RNA and protein assay.

### Lentiviral vector construction, packaging and lateral cerebroventricular administration

Full length cDNA of wildtype (wt) Mkrn3 and Nptx1 was cloned from mouse brain tissues, labeled with Flag tag and constructed into FUGW vector (Invitrogen) contained co-expressed GFP. Nptx1 was labeled with flag at N- or C-terminal region respectively. The artificial mutation (mt) vectors were obtained based on wildtype vector using mutagenesis PCR. All primers used for this study are listed in Table 1. Lentiviral packaging and titer determination were performed as previously described [3]. Briefly, the virus preparation system comprised vector (16 μg), Δ 8.9 (12 μg), and VSVG (8 μg) were transfected into 293T cells for 48 h. The supernatant was harvested, gradient diluted and evaluate the titer determination via counting the number of fluorescent cells with GFP. 3-week old mice were anesthetized to fixed on stereotaxic apparatus, incised 1cm of the fur and skin along the head mid-line and scraped periosteum to expose the Bregma point. A microsyringe was used to pierce the skull at a 90° angle, at the point 1.0 mm from the posterior Bregma point and 0.5 mm to the right of the midline. The needle was inserted into a depth of 3.5 mm (A-1.0, R0.5, H3.5) and retained before and after 2×10^8^ v.p (50ul) virus injection for 5-10 min, then removed. The PBS was used for administration as the negative control. Finally, the skin was sewn up. The mice were not sacrificed until 4-, 6- and 10- week old for hypothalamus harvest.

**Table 1:** All primers used in this study are listed

Gene symbol	Primer sequence	Tm (°C)
wtMkrn3	F: 5′-ATGGAAGAGTCTACAGCTCCCATTG-3′	58
	R: 5′-CTACAGATTCAAACTGAAATATTCTTCC-3′	
mtMkrn3 (347-401 deletion)	F: 5′-CGCAGCATGGACAAAGTGGTCTCCTCTGGCTTTGTC-3′	50
	R: 5′-GACAAAGCCAGAGGAGACCACTTTGTCCATGCTGCG-3′	
wtNptx1	F: 5′-ATGCTGGCCGGCCGC-3′	63
	R: 5′-TCAGTTGATCTGGCGACAAGCCTC-3′	
mtNptx1 (268-302 deletion)	F: 5′-TGCGGCGCCTGGAGTGCTGCCGTTTGTAATCAACG-3′	52
	R: 5′-CGTTGATTACAAACGGCAGCACTCCAGGCGCCGCA-3′	

### Immunoprecipitation (IP) and immunoblotting

The hypothalamus tissues were pestled in mortar with liquid nitrogen, added 1ml IP lysis buffer (50mM NaCl, 50mM Tris, [pH 7.4], 1% NP-40) with protease inhibitor, and transferred into 1.5ml tube after thawing. The cell lysates were mixed with 1ug Flag antibody (Invitrogen, Catalog No. MA1-142) and 40ul of flurry protein A beads (Invitrogen, Catalog No. 101141) for rotating overnight at 4°C. The immunoprecipitates were washed three times with washing buffer contained 250mM NaCl. Eluate from IP-pellets was then western blotted for appropriate antibodies. Rabbit IgG was used as negative control. For immunoblotting, briefly, the transfer-ready membrane was blocked overnight in TBST (10 mM Tris-HCl [pH 7.5], 150 mM NaCl, 0.1% Tween-20) contained 5% nonfat milk at 4°C, followed by incubation with appropriate primary antibody of Mkrn3 (Abcam ab174878) or Nptx1 (Santa cruz, sc374199). The secondary antibodies were horseradish peroxidase-conjugated anti-mouse (sc2005), anti-rabbit (sc2357) antibodies were used at a 1:5000 dilution. Gapdh (Santa cruz, sc47724) was used as a loading control. All the experiments were repeated 5 times for biological repetition.

### Proteins modeling and docking

The protein structures of mouse Nptx1 and Mkrn3 were modeled in I-TASSER server (http://zhanglab.ccmb.med.umich.edu/I-TASSER/) [30], and molecular docked in Z-DOCK server (http://zdock.umassmed.edu/) [31]. The interacted domain was picked up using pymol from the given top models according to the experimental data.

## SUPPLEMENTARY MATERIALS FIGURES


