# Supplementary material for: Mkrn3 functions as a novel ubiquitin E3 ligase to inhibit Nptx1 during puberty initiation
Source: Oncotarget. 2017 Jul 18;8(49):85102–9. doi: 10.18632/oncotarget.19347 (PMC5689596; doi:10.18632/oncotarget.19347)
Supplement: Supplementary file 1 [file oncotarget-08-85102-s001.pdf]

Mkrn3 functions as a novel ubiquitin E3 ligase to inhibit Nptx1 during puberty initiation

SUPPLEMENTARY MATERIALS

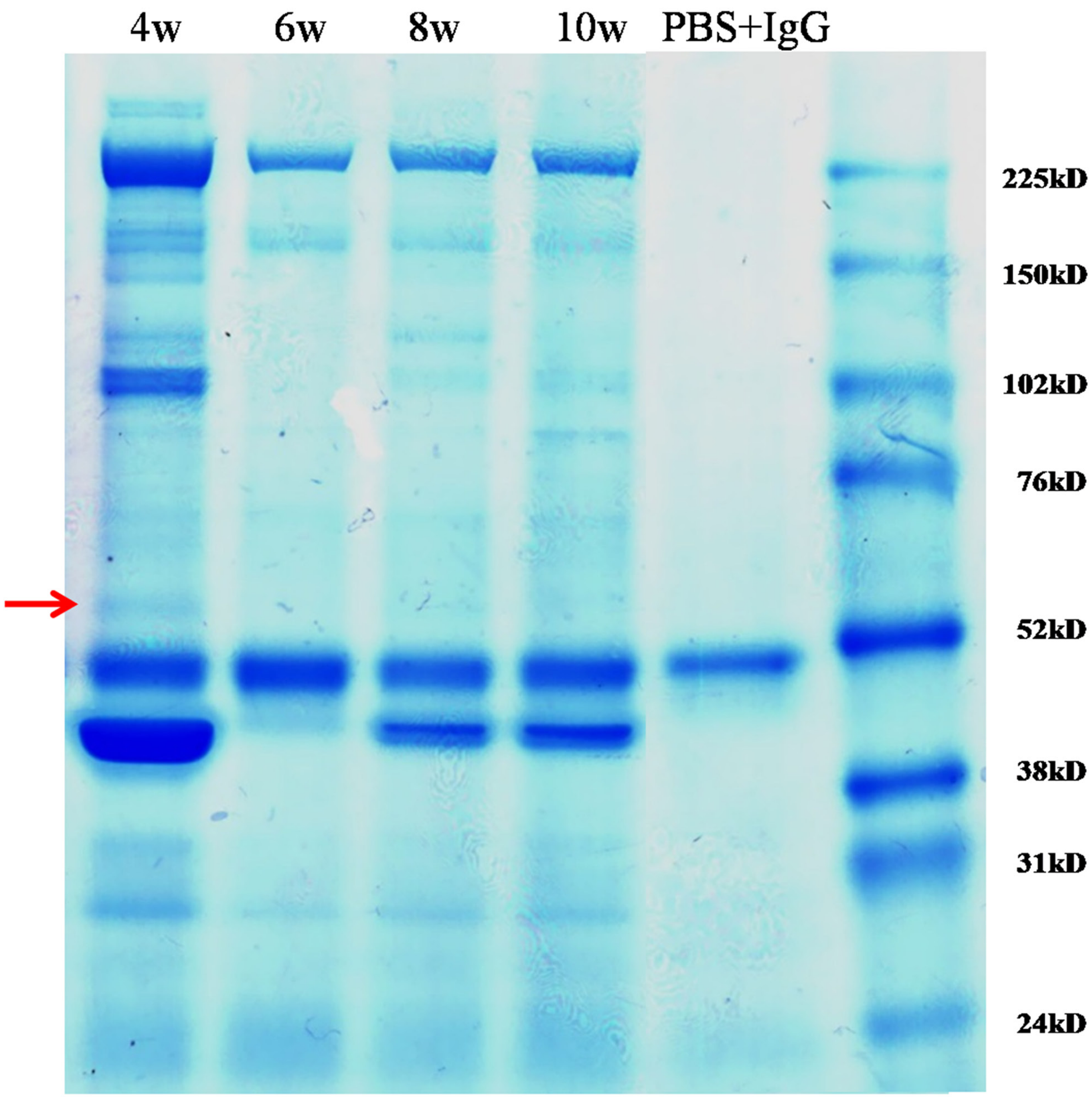

Supplementary Figure 1: The blue staining of protein pattern from 4-, 6-, 8-, and 10-week old mouse hypothalamus pulled down by Nptx1 antibody. The band marked with red arrow was identified as Mkrn3 by mass spectrometry in 4-week old hypothalamus. Mouse IgG was used as a negative control.

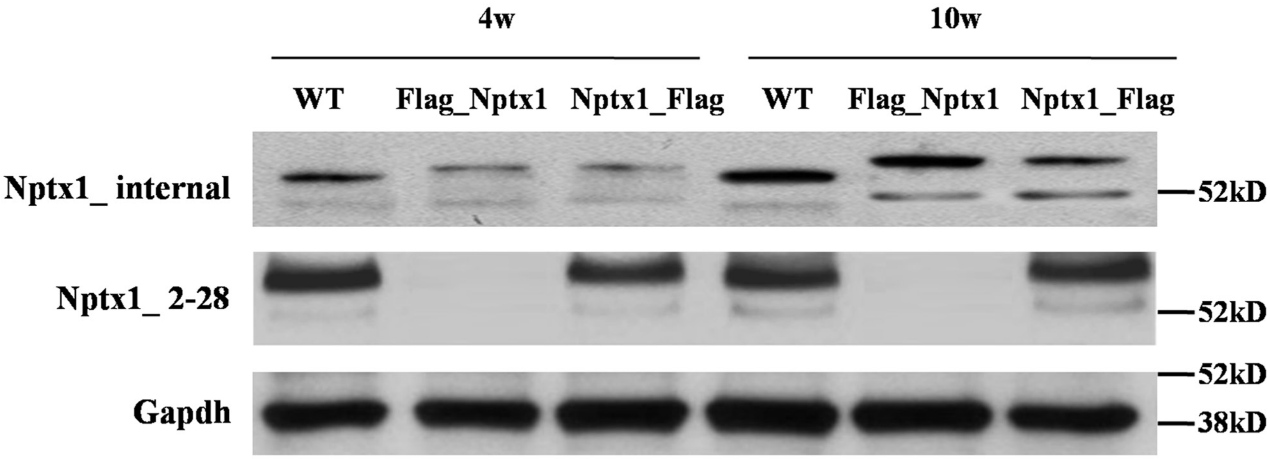

Supplementary Figure 2: Identification of the signal peptide of Nptx1. Nptx1 protein was investigated by antibody of Nptx1 (Nptx1_internal against the internal region while Nptx1_2-28 against the N-terminal region). Flag_Nptx1 and Nptx1_Flag represent three flags around 22 amino acids inserted into N-terminal and C-terminal fusion protein respectively.

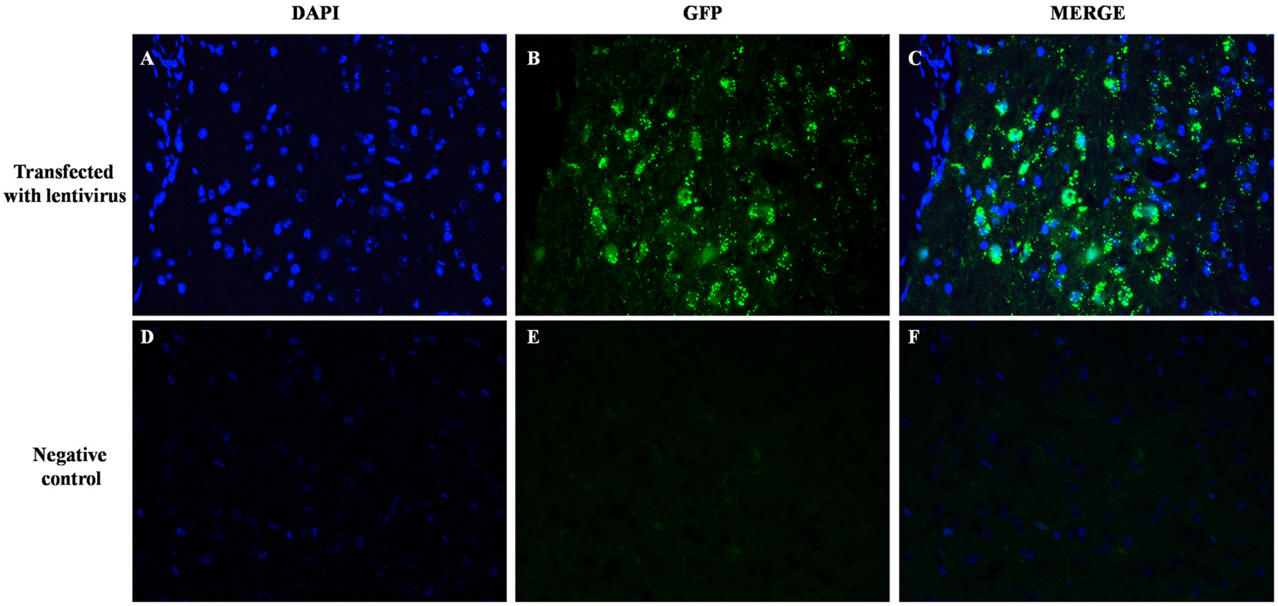

Supplementary Figure 3: The efficiency of cerebroventricular administration. GFP and flag labeled exogenous genes (Nptx1 or Mkrn3) are co-expressed in all our vectors. GFP positive cells represent that those cells have been transfected with lentivirus **(B)** compared to control **(E)**. DAPI staining is used to mark the nucleus **(A, D)** and merged with GFP **(C, F)**.

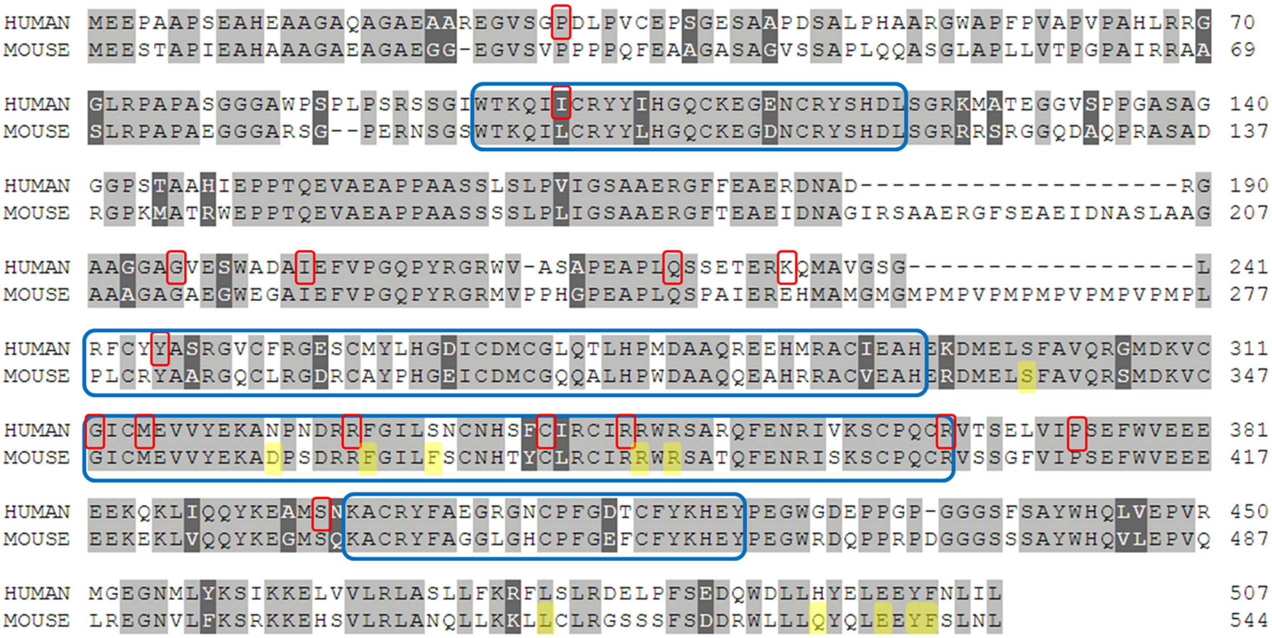

Supplementary Figure 4: The amino acid sequence alignment of human and mouse Mkrn3. Disordered regions are indicated as dashed lines. The same residues are colored as light gray, the residues having similar polarity as dark gray. The missense mutations in human are framed as red. The residues of C3H1 motif and C3H4 Ring finger domain are framed as blue. The directly interacted sites of Mkrn3 with Nptx1 are labeled as yellow.
